# A feasibility study evaluating a reservoir storage system for continuous oxygen delivery for children with hypoxemia in Kenya

**DOI:** 10.1186/s12890-021-01433-6

**Published:** 2021-03-05

**Authors:** Dickson Otiangala, Nicholas O. Agai, Bernard Olayo, Steve Adudans, Chin Hei Ng, Ryan Calderon, Ella Forgie, Christine Bachman, Daniel Lieberman, David Bell, Michael Hawkes, Akos Somoskovi

**Affiliations:** 1 Center for Public Health and Development, Nairobi, Kenya; 2 Intellectual Ventures Laboratory, Bellevue, WA USA; 3 Intellectual Ventures, Global Good Fund, Bellevue, WA USA; 4 Department of Pediatrics, University of Alberta, 3-588D Edmonton Clinic Health Academy, 11405 87 Ave NW, Edmonton, AB T6G 1C9 Canada; 5 Department of Medical Microbiology and Immunology, University of Alberta, Edmonton, Canada; 6 Department of Global Health, School of Public Health, University of Alberta, Edmonton, Canada; 7 Stollery Science Lab, University of Alberta, Edmonton, Canada; 8 Women and Children’s Health Research Institute, University of Alberta, Edmonton, Canada; 9 Present Address: Issaquah, WA USA

**Keywords:** Pneumonia, Hypoxemia, Electricity, Global health, Africa

## Abstract

**Background:**

Supplemental oxygen is an essential treatment for childhood pneumonia but is often unavailable in low-resource settings or unreliable due to frequent and long-lasting power outages. We present a novel medium pressure reservoir (MPR) which delivers continuous oxygen to pediatric patients through power outages.

**Methods:**

An observational case series pilot study assessing the capacity, efficacy and user appraisal of a novel MPR device for use in low-resource pediatric wards. We designed and tested a MPR in a controlled preclinical setting, established feasibility of the device in two rural Kenyan hospitals, and sought user feedback and satisfaction using a standardized questionnaire.

**Results:**

Preclinical data showed that the MPR was capable of bridging power outages and delivering a continuous flow of oxygen to a simulated patient. The MPR was then deployed for clinical testing in nine pediatric patients at Ahero and Suba Hospitals. Power was unavailable for 2% of the total time observed due to 11 power outages (median 4.6 min, IQR 3.6–13.0 min) that occurred during treatment with the MPR. Oxygen flowrates remained constant across all 11 power outages. Feedback on the MPR was uniformly positive; all respondents indicated that the MPR was easy to use and provided clinically significant help to their patients.

**Conclusion:**

We present a MPR oxygen delivery device that has the potential to mitigate power insecurity and improve the standard of care for hypoxemic pediatric patients in resource-limited settings.

**Supplementary Information:**

The online version contains supplementary material available at 10.1186/s12890-021-01433-6.

## Background

Pneumonia accounts for more fatalities in children globally than any other infectious disease [1]. In 2010, 120 million cases of pneumonia were recorded in children under 5 years of age. Treatment includes supplemental oxygen [2]; however, the availability of medical oxygen is unreliable in many low- to middle-income countries (LMICs) [3]. Our study took place in Kenya, a high burden country for pediatric pneumonia, with widespread oxygen insecurity. The under-five mortality rate in Kenya is approximately 4.6%, with 68,882 deaths annually [1]. Pneumonia accounts for 16% of child mortality, the second leading cause of death in children [2].

Hypoxemia, a blood oxygen saturation (SpO_2_) < 90%, increases the risk of mortality in children with pneumonia fivefold [4, 5]. Therefore, timely diagnosis and treatment of hypoxemia are essential to optimize patient outcomes [6]. Oxygen cylinders and oxygen concentrators are standard oxygen delivery methods employed in LMICs [6, 7]. However, cylinders are costly to transport, require regular replenishment [8], and are only feasible as a method of oxygen delivery where there is a reliable supply chain [9]. Oxygen concentrators [10] are less costly and more convenient than cylinders; however, they depend on a reliable source of electricity [9].

Power insecurity in healthcare facilities in LMICs remains a critical barrier to providing reliable oxygen delivery [11]. Power interruptions in many areas are frequent and long-lasting [3]. One solution may be to store oxygen in a reservoir while power is available, for use during power outages. Currently, there are a limited number of devices capable of bridging power outages to deliver continuous oxygen [6, 7, 12]. High-pressure oxygen storage systems are associated with safety hazards and may be vulnerable to leakage from fittings. On the other hand, low pressure reservoirs (LPRs) occupy a large volume and have restricted mobility [12]. LPRs are often housed outside of the ward and may not be feasibly installed in all facilities [13]. A medium-pressure reservoir (MPR) may have the benefits of safety, ruggedness, and compact size.

The objectives of our study were: (1) to design and test an MPR in a controlled pre-clinical environment; (2) to establish the feasibility of using the MPR device in two low-resource hospitals; (3) to determine whether the MPR was capable of delivering an uninterrupted oxygen supply during power outages; and (4) to assess end-user (health worker) feedback on the MPR oxygen delivery system.

## Methods

### Pre-clinical design

The design of the MPR sought to balance the need for continuous supply of oxygen with the cost, complexity, and ease of use of the storage device. An ideal MPR solution would maximize stored oxygen volume while minimizing the system’s physical footprint and operational complexity while being compatible with all commercial oxygen concentrators. Considerations included in the design were: (1) preference for off-the-shelf commercial products to capitalize on economies of scale costing and manufacturer component durability testing; (2) appropriate boost compressor size so it was not choked by output specifications of the oxygen concentrator and would reliably operate up to our maximum pressure without stalling; (3) safety of operation with respect to ignition risk due to storing high concentration oxygen at elevated pressure. A more complete description of the design considerations is included in the online supporting materials.

### Clinical pilot study design and setting

This was an observational case series piloting the MPR on a small number of children with hypoxemia. Two rural hospitals in Kenya were chosen based on their high volumes of pediatric inpatients and unreliable electricity for deployment of the MPR oxygen delivery system: Ahero Hospital (Kisumu County), and Suba Hospital (Homa Bay County). We have previously reported on the availability and reliability of medical oxygen and electrical power at these and other facilities in the area [3].

### Clinical definitions

Tachypnea and tachycardia were defined as respiratory rate or heart rate above the 99th percentile for age, respectively [14]. The Signs of Inflammation in Children that Kill (SICK) score is a composite severity-of-illness instrument used to predict the risk of subsequent mortality [15]. Non-invasive clinical assessment of patients’ respiratory rate, heart rate, capillary refill time, SpO_2_, systolic blood pressure and temperature are used to calculate the SICK score [16]. Patients with SICK scores exceeding 2.3 are at high risk of mortality [17].

### Inclusion, exclusion and early discontinuation criteria

Patients were enrolled in the study if they met the following inclusion criteria: (1) age > 1 year and < 16 years; (2) hypoxemia (SpO_2_ < 90%); (3) admission to hospital warranted by the attending physician; and (4) provided written informed consent from the child’s legal guardian, and assent from children > 7 years of age. Exclusion or early discontinuation from the MPR occurred if: (1) patient SICK scores exceeded 2.3; (2) they required an oxygen flowrate > 2 L/min; or (3) an alarm indicated that reservoir was nearly depleted or oxygen concentration was < 82%.

### Equipment and patient monitoring

The MPR instrument was monitored using near-continuous measurements of the flowrate (Honeywell Zephyr, Charlotte, NC), tank pressure (Honeywell PX3, Charlotte, NC), and oxygen quality (Compass Controls Manufacturing, 120-G, Lenexa, KS). Grid power reliability was assessed using a customized enVision 23,010 IC power monitor (Ametek®, Knightdale, NC). Measurements were logged every 0.1 min and stored for future analysis at the end of the trial. Nurses monitored patient vital signs as well as oxygen saturation every four hours (Rad-5® oximeter, Masimo Corp., Irvine, CA).

### Satisfaction questionnaire

A 10-item satisfaction questionnaire was designed to systematically evaluate the user feedback on the device. The questionnaire was administered to a convenience sample of users of the device at the end of the clinical trial and included information on ease of use, perceived strengths and limitations, and overall appraisal of the MPR.

## Results

### Design and pre-clinical testing of medium pressure reservoir

**Fig. 1:**
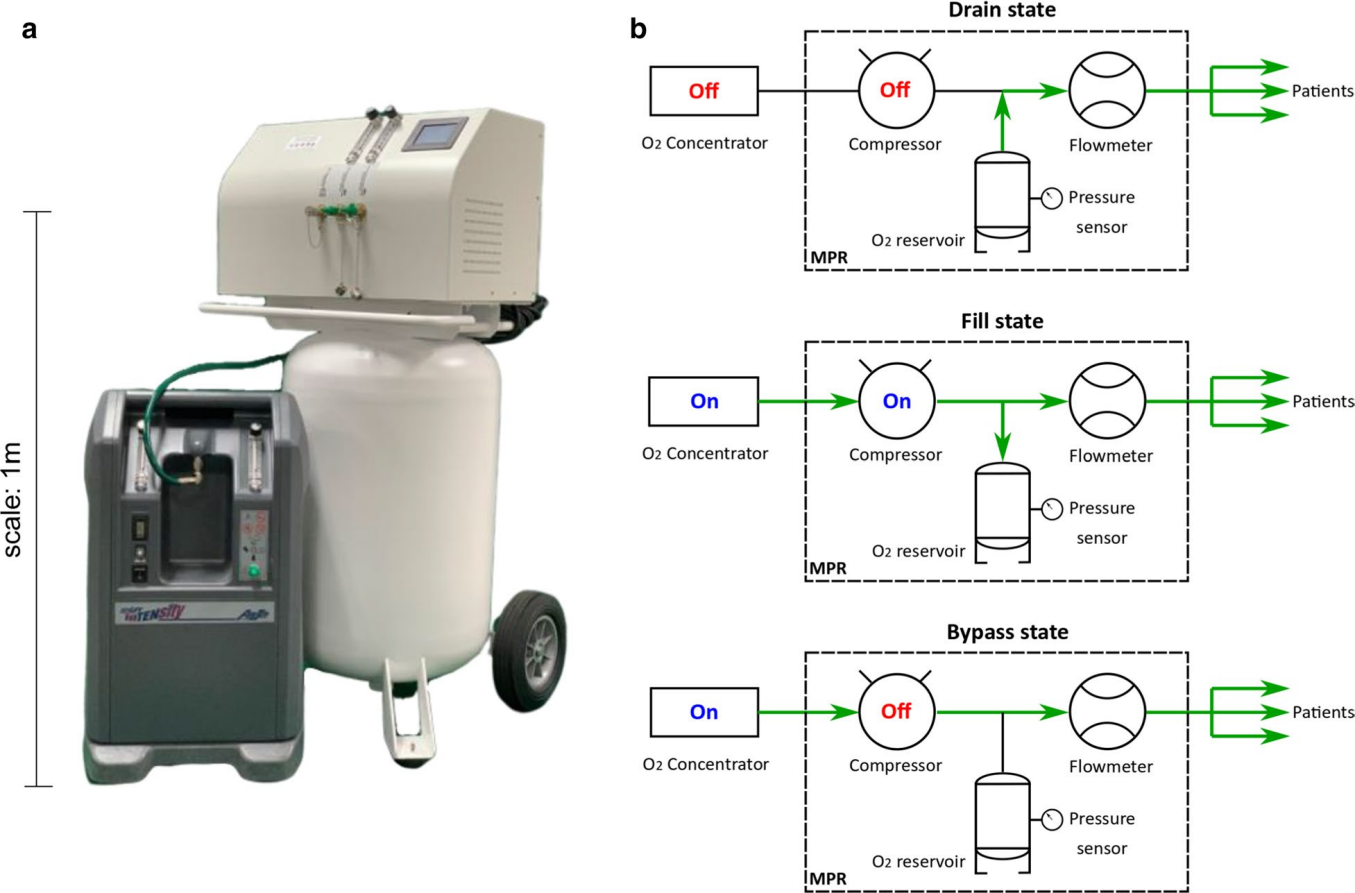
Medium pressure reservoir (MPR) to bridge gaps in oxygen delivery. **a** Picture of the MPR. **b** Functional states of the MPR. Drain state represents the power outage scenario where oxygen is drawn from the oxygen reservoir to patients. Fill state represents the normal operating condition (power available) where input from the oxygen concentrator is used to fill the reservoir and/or directed to patients. Bypass state is used when filling the reservoir is not a priority (reservoir is full or patient demand is high). In this state, oxygen is drawn directly from the oxygen concentrator to patient

**Fig. 2:**
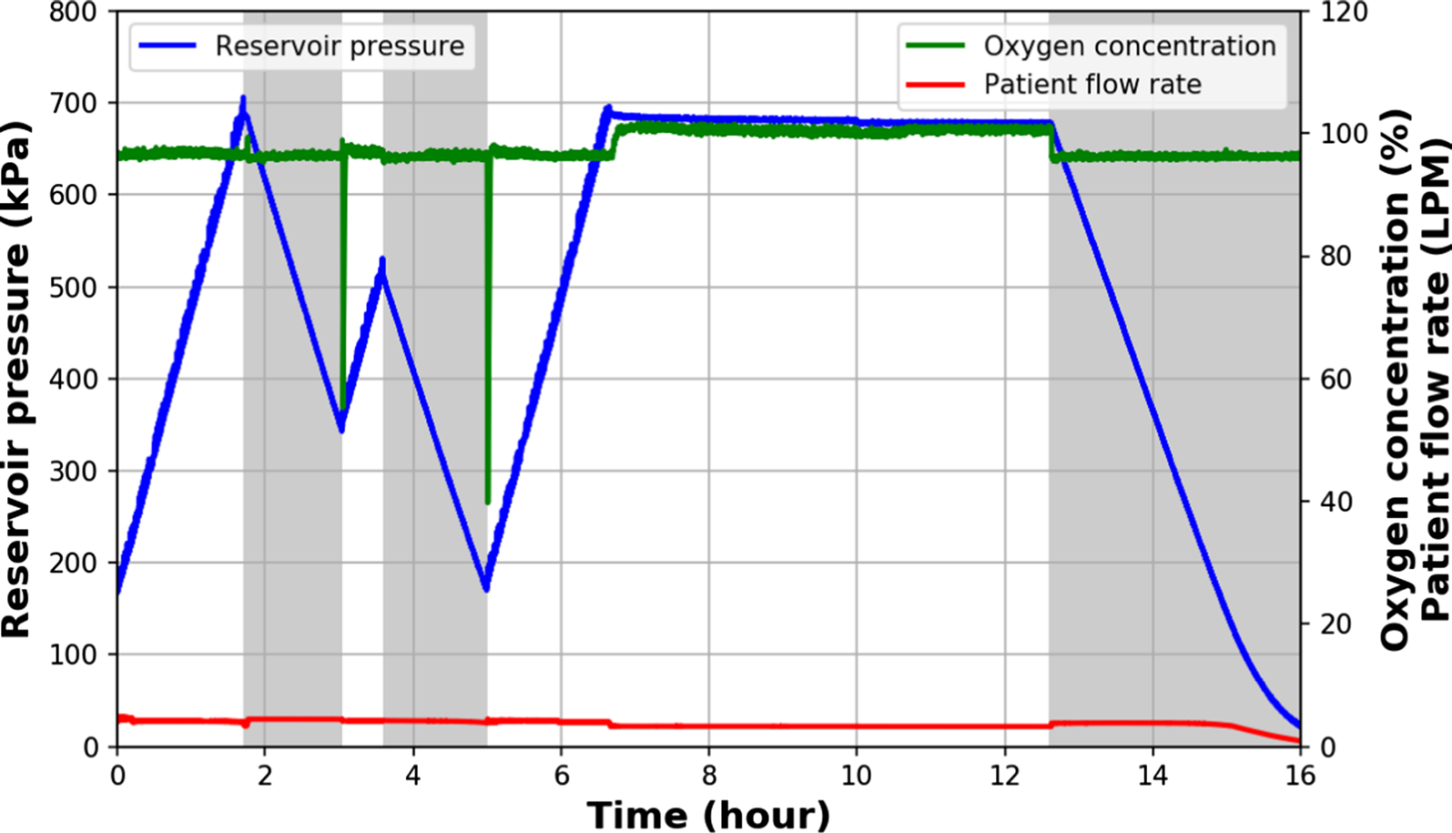
Pre-clinical testing of medium pressure reservoir (MPR). Benchtop performance of the MPR with simulated power outages (shaded region) and a constant patient flowrate of 5 L/min

We designed a novel MPR that integrates with a commercial oxygen concentrator (Airsep Newlife Intensity 10, Chart Industries, Ball Ground, GA) to provide a continuous stream of oxygen during power interruptions (Fig. 1a, Additional file 1: Figure S1, Table S1). This device capitalized on power when it is available, providing oxygen to up to two patients at a time while simultaneously diverting excess oxygen to a reservoir (Fill state, Fig. 1b). In the event of a power interruption, the device automatically began delivering stored oxygen to the patients (Drain state, Fig. 1b). Alternatively, the device could be operated in Bypass state (Fig. 1b), when the reservoir is full or patient demand is high. In pre-clinical testing, the fully pressurized MPR delivered a continuous stream of oxygen at a clinically relevant flowrate of 5 L/min for approximately 3 h under conditions of simulated power outages (Fig. 2). The MPR stored oxygen up to 790 kPa and could provide continuous oxygen flow for 891, 445, and 178 min at 1, 2, and 5 LPM flowrates, respectively. The MPR was designed and manufactured to comply with relevant safety standards to meet the objectives of this study [18–22].

### Pilot clinical trial

Between May 20, 2019 and July 10, 2019, 88 pediatric patients hospitalized at Ahero and Suba Hospitals were screened. Nine met inclusion criteria and were enrolled in a pilot study of the MPR (Additional file 1: Figure S2). Patient characteristics are shown in Table 1.

**Table 1:** Characteristics of trial participants

Characteristic	Summary statistic (N = 9)
Demographics	
Female sex, n (%)	4 (44)
Age (years), median (IQR)	2 (1–3)
Facility, n (%)	
Suba	5 (56)
Ahero	4 (44)
Vital signs at admission	
Temperature (axillary, °C), median (IQR)	37.3 (36.7–37.7)
Fever (Tax > 37.5 °C), n (%)	4 (44)
Respiratory rate (breaths/min), median (IQR)	38 (38–42)
Tachypnea,^a^ n (%)	5 (56)
Heart rate (beats/min), median (IQR)	136 (119–149)
Tachycardia,^a^ n (%)	3 (33)
Systolic blood pressure (mmHg), median (IQR)	95 (90–98)
Hypotension, n (%)	0
SpO_2_ (% on room air),^b^ median (IQR)	80 (79–85)
Hypoxemia (< 90%), n (%)	9 (100)
History of presenting illness, n (%)	
Cough	8 (89)
Difficulty breathing	9 (100)
Convulsions	1 (11)
Not eating/drinking anything	1 (11)
Physical exam at admission	
Chest indrawing, n (%)	7 (78)
Wheezing, n (%)	1 (11)
Delayed capillary refill time (> 3 s), n (s)	0
Altered consciousness, n (%)	0
Composite clinical severity (SICK) score,^c^ median (IQR)	1.7 (1.7–1.9)
Diagnoses, n (%)	
Pneumonia	7 (78)
Malaria	3 (33)
Other^d^	3 (33)
MPR utilization, n (%)	
Patient finished O_2_ treatment with MPR	5 (56)
MPR stopped early, switch to O_2_ cylinder	4 (44)
Outcome, n (%)	
Discharged home	5 (56)
Transferred to another facility	3 (33)
Fatal	1 (11)

^a^Defined as > 99th percentile for age [14]

^b^Peripheral blood oxygen saturation

^c^Signs of Inflammation in Children that Kill (SICK) [14, 15]

^d^One patient was diagnosed with HIV, tuberculosis and malnutrition; one patient was diagnosed with sickle-cell disease; and one patient was diagnosed with diarrhea and convulsions

**Fig. 3:**
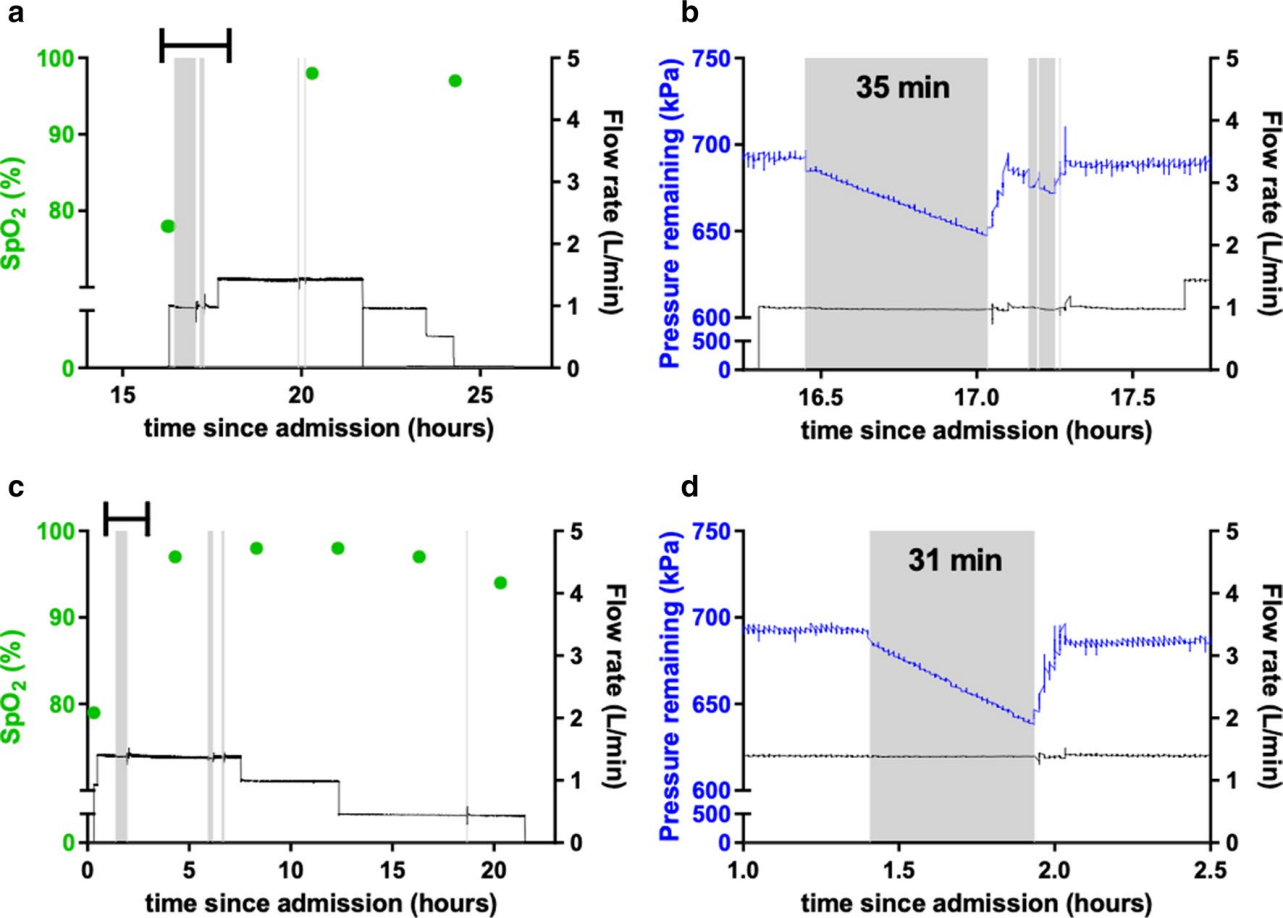
The MPR bridges power failures in Kenyan children receiving oxygen therapy. **a**, **c** In two representative patients, oxygen flowrate (black line) and peripheral oxygen saturation (spot check, green circles) were maintained through several power cuts, including cuts lasting > 30 min. **b**, **d** MPR output flowrate (black line) and tank pressure (blue line) through the longest power cuts. Flow was maintained during the power cut. The MPR pressure dropped as expected during power cut and rose again with tank filling when power returned

Patients required oxygen therapy for a median duration of 9.2 h (range 1.1–43), representing a total time of oxygen delivery of 162 patient-hours. A total of 11 power interruptions lasting more than one minute (median duration 8.2 min, IQR 4.8–13) were observed, representing 1.4% of the total time of oxygen delivery (Table 2). The two longest power outages (34.7 min and 31.2 min) are depicted in Fig. 3. Oxygen flowrates did not change when power outages occurred.

**Table 2:** Power failures during study period

Outage #	Facility	Patient #	Duration of outage (min)	Flow rate 5 min prior to outage, mean (SD) (L/min)	Flow rate during the outage, mean (SD) (L/min)
1	Suba	1	34.7	0.99 (0.0071)	0.97 (0.0072)
2	Suba	2	31.2	1.39 (0.010)	1.38 (0.0042)
3	Suba	2	13.4	1.36 (0.010)	1.35 (0.0064)
4	Suba	3	12.5	1.50 (0.012)	1.08 (0.66)^3^
5	Suba	4	8.7	1.65 (0.012)	0.14 (0.43)^c^
6	Ahero	5	8.2	1.20 (0.011)	1.45 (0.0046)
7	Suba	6	7.9^a^	0.0082 (0.0059)^b^	0.93 (0.21)^b^
8	Suba	1	5.0	0.99 (0.025)	0.98 (0.017)
9	Suba	2	4.6	1.37 (0.011)	1.36 (0.012)
10	Suba	4	4.5	0.55 (0.0025)	0.55 (0.080)
11	Suba	3	4.3	1.51 (0.015)	1.49 (0.014)

^a^The power was inconsistent for 12.1 min, including three periods without power lasting 2.2, 1.5 and 4.2 min, and two periods in which the power briefly resumed for 1.3 and 2.9 min

^b^Oxygen therapy was started during the power outage

^c^Oxygen therapy was stopped during the power outage

Four patients (44%) were removed from the MPR device early and transferred to a standard oxygen cylinder (Table 1, Additional file 1: Figure S2) for the following reasons: they required a higher oxygen flowrate than mandated by the study protocol (> 2 L/min); an alarm signaled that oxygen purity had fallen below an acceptable standard; they needed to be transferred to another facility; or they were uncooperative. One patient in our study presented with hypoxemia, malaria, and stridor at rest, and subsequently died (11%). Careful review of the case concluded that death was unrelated to the MPR device for the following reasons: (1) the patient was transferred to a standard oxygen cylinder one hour prior to death, per protocol, because the required flowrate was greater than the protocol-mandated maximum of 2 L/min; (2). no significant power outages (> 1 min) were recorded, therefore the reservoir tank was never utilized; and (3) oxygen purity from the concentrator remained at > 82% throughout treatment.

### End user satisfaction questionnaire

Seven users (nurses and clinicians) from Suba Hospital provided feedback on the MPR at the end of the trial, using a standardized satisfaction questionnaire (Table 3).

**Table 3:** End user satisfaction questionnaire

Questionnaire item	Percent agreement^a^ (N = 7)
The MPR is easy to use	7 (100)
It is difficult to…	
Turn on and off	0
Monitor the level of O_2_ and alarm signals during power outages	0
Adjust the flow rate	0
The back-up O_2_ provided clinically significant help during power outages	7 (100)
The best features of the device are…	
O_2_ remainder indicator	4 (57)
Automated transition to back-up O_2_ cylinder during power outages	3 (43)
Ability to treat 2 patients at once	2 (29)
Ability to deliver continuous O_2_ through power outages	7 (100)
Capacity to cover power outages	6 (86)
Features that need to be improved are…	
O_2_ remainder indicator and alarms	0
Capacity of the MPR to treat more patients	3 (43)
O_2_ purity indicator and alarms	1 (14)
Capacity of the MPR to provide O_2_ for longer durations	5 (71)
Footprint of the MPR	0
During a power outage, the MPR…	
Worked without the need to make adjustments in O_2_ therapy	4 (57)
Worked with minimal control of monitoring the amount of stored O_2_	3 (43)
Worked without the need to make adjustments in O_2_ therapy based on the remaining O_2_	2 (29)
Was not used to significantly improve uninterrupted O_2_ supply	0
The use of the MPR during a power outage interrupted typical standard of care	0
It is easy to move the concentrator linked to the MPR within the room	4 (67)^b^
The location of the storage tank is a disturbance to daily work and facility management	0^c^
You would change the size of the MPR	2 (33)^b^
You would change the location of the MPR	0^b^

^a^For Likert scale questions, agree or strongly agree were considered agreement. For yes/no questions, affirmative response was considered agreement

^b^One respondent answered "not applicable" and was not included in the calculations

^c^On a Likert scale from 1 to (no disturbance to significant disturbance), six respondents answered 1 and one respondent answered 2

## Discussion

Here we demonstrate the potential utility of a novel MPR that can bridge power outages to deliver a continuous oxygen supply. After pre-clinical development and testing, the MPR was deployed in two rural hospitals in Kenya, demonstrating feasibility for use on resource-limited pediatrics wards. Real-world power outages lasting > 30 min were seamlessly and automatically bridged by the MPR. User feedback was uniformly positive. Our study supports further development of the MPR device and encourages larger non-inferiority trials.

Hypoxemia is a severe and life-threatening complication of pneumonia. The median prevalence of hypoxemia in children with pneumonia requiring hospitalisation is 13% [23]. The case-fatality rate of pneumonia ranges from 3 to 15%, with a fivefold higher odds of death in children with hypoxemia [24]. In our study, 9/88 (10%) of screened patients were hypoxemic, and 1/9 (11%) of hypoxemic patients died, consistent with these data.

More than half of the patients in our study experienced one or more power outages, all of which were successfully bridged by the MPR (Table 2). Most power outages occurred at a critical time of oxygen dependency, such that clinical consequences may have been severe without backup flow from the MPR. Two power interruptions lasted > 30 min and were potentially life-threatening. A previous study on power insecurity in 12 sub-Saharan African countries found that electricity was fully available in only 35% of the 231 healthcare facilities assessed [25]. We previously showed that power was unavailable for a median of 7% of the time in Kenyan facilities [3]. In light of these findings, tenuous electrical supply in our study and previous studies highlights the need for interventions like the MPR.

Previous studies have assessed the use of high-pressure and low-pressure oxygen storage reservoirs in pre-clinical studies [12, 13, 26]. In hospitals where there is a reliable supply chain from an oxygen plant, high-pressure oxygen cylinders are the standard. However, a previous study in Uganda found that only 10% of nurses showed adequate skills in operating a high-pressure oxygen cylinder [26] and tightening of high-pressure mechanical fittings requires considerable manual strength [27]. Our MPR was user-friendly and did not require manual intervention from the ward nurses. Problems with low-pressure oxygen cylinders are associated with the size of the reservoir. Space requirements are considerable, and often lead to the relocation of the low-pressure reservoir outside the hospital ward [13]. In contrast, the MPR was maneuverable and integrated well in the clinical workflow. Thus, medium-pressure oxygen storage methods have the potential to overcome many of the limitations associated with high- and low-pressure oxygen cylinders.

There are several limitations to our study. The design was a pilot study that did not have a control group and was not statistically powered to demonstrate efficacy of the MPR. Our conclusions are therefore limited to feasibility of the MPR and its ability to bridge power outages on resource-limited pediatrics wards. We included two health facilities; however, a wider sampling of hospitals and health centres, across a range of available grid electricity, would be desirable to assess the ideal use case for the MPR. The cut off for the SICK score excluded the sickest patients and limited our ability to know how this intervention could impact outcomes in that cohort. Our study was limited to public facilities whereas non-state providers are increasingly present in low-income settings; our conclusions should therefore be extrapolated with caution to private health care facilities. The device described in this study is an engineering sample designed to test the feasibility of providing continuous oxygen through power outages. A commercial product is being developed with cost appropriate to LMICs being a consideration. The return on investment over the lifetime of the device should be comparable to the cost of oxygen cylinders and the logistics to deliver them. A formal cost-effectiveness analysis would be of interest, but is beyond the scope of the current manuscript.

## Conclusion

Our MPR mitigates the risks of power insecurity and may improve the standard of care for hypoxemic pediatric patients in resource-limited settings. This device is likely to have the highest impact in high-volume health centers with frequent power interruptions, and it may not be suitable for large district hospitals. Further studies into where the MPR proves most effective are warranted.

## Supplementary Information

**Additional file 1.** Materials provided in the online additional file include: (1) Engineering design of medium pressure reservoir (MPR); (2) **Figure S1**, Detailed schematic of MPR; (3) **Table S1**, Description of components of MPR; and (4) **Figure S2**, Pilot clinical trial profile.

## Data Availability

The datasets used and/or analyzed during the current study are available from the corresponding author on reasonable request. The data are available at the following clinical data repository: Association for Health Innovation in Africa (AFHIA, https://sites.google.com/view/afhia-drc-uganda/clinical-data-repository. Accession number 2020.001).

## Abbreviations

LMICs: Low-to middle-income countries
SpO_2_: Blood oxygen saturation
LPRs: Low-pressure reservoirs
MPR: Medium-pressure reservoir
SICK: Signs of inflammation in children that kill
