# Supplementary material for: A feasibility study evaluating a reservoir storage system for continuous oxygen delivery for children with hypoxemia in Kenya
Source: BMC Pulm Med. 2021 Mar 5;21:78. doi: 10.1186/s12890-021-01433-6 (PMC7934496; doi:10.1186/s12890-021-01433-6)
Supplement: Supplementary file 1 — Additional file 1. Materials provided in the online additional file include: (1) Engineering design of medium pressure reservoir (MPR); (2) Figure S1, Detailed schematic of MPR; (3) Table S1, Description of components of MPR; and (4) Figure S2, Pilot clinical trial profile. [file 12890_2021_1433_MOESM1_ESM.docx]

**Additional file**

**A feasibility study evaluating a reservoir storage system for continuous oxygen delivery for children with hypoxemia in Kenya**

Dickson Otiangala, MPH; Nicholas O. Agai, MBChB, MMed; Bernard Olayo, MBChB, MPH; Steve Adudans, MBChB, MSc; Chin Hei Ng, PhD; Ryan Calderon, BEng; Ella Forgie, BSc; Christine Bachman, MPH; Daniel Lieberman, PhD, PE; David Bell, MBBS, PhD; Michael Hawkes, MD, PhD; Akos Somoskovi, MD, PhD, DSc

The Global Good/Intellectual Ventures’ MPR storage system was designed to operate on patients in a clinical trial setting where the intended use is to provide oxygen or alarm. The MPR system

consists of a custom-made stainless-steel reservoir on a mobile wheelbase and oxygen flow control user interface. A schematic of the MPR is shown in E-Figure 1A. The MPR is designed to be compatible with a commercial oxygen concentrator (e.g., Airsep Newlife Intensity 10, dual output) and together form an oxygen generation and storage system to provide a continuous stream of oxygen during power interruptions. Remaining oxygen capacity is shown in the built-in display during the power failure. An alarm alerts the user when the reservoir is nearly depleted or oxygen concentration is below 82%. The device functions differently under two scenarios: power available and power outage. When electrical power is available, oxygen is provided to patients while simultaneously diverting excess oxygen to the reservoir (Fill state, E-Figure 1B). In the event that the reservoir is full or that patient demand is higher than oxygen inlet flow rate, the compressor is shut off and oxygen from the concentrator(s) flows through a bypass check valve directly to the outlets bypassing the internal compressor and storage (Bypass state, E-Figure 1B). In the event of an electrical power interruption, the device automatically begins delivering stored oxygen to the patients (Drain state, E-Figure 1B). By design, oxygen flow to the patient(s) is prioritized over oxygen flow into the storage vessel.

*Components*

Internally, the MPR includes a boost compressor, automated flow control, pressure sensors, oxygen quality sensor, temperature and humidity sensors, exhaust fans, data recording, and GSM communication to cloud-based database; all in a field serviceable housing. Externally, there are three DISS connectors with two for direct patient flow control via the ball-in-tube rotameter (10 LPM range) and the third for routing to external flow splitter (output pressure 16 PSI), oxygen concentrator electrical connection inside a recessed vault, and power control switch. MPR device is designed to fill the storage reservoir with excess oxygen that is generated by the oxygen concentrator, while being able to provide up to 20 L/min for all outlet ports combined. The reservoir can store up to 786 L of O2 with a maximum operating pressure of 690 kPa. This corresponds to approximately 6.5 hours of oxygen delivery at 2 L/min. The MPR can control two 550W oxygen concentrators for a combined input oxygen flow rate of 20 L/min.

The MPR was designed and manufactured with strict requirements to reduce ignition risk due to storing high concentration oxygen at elevated pressure. First, all wetted components were selected to be compatible with oxygen and cleaned to the level specified in an oxygen safety standard (ASTM G93) prior to assembly (hydrocarbons < 550 mg/m^2^ and particulate level 300).^1^ Second, inline filters were installed to minimize particulates in the flow circuit. High power electronics were isolated from the flow circuit in order to separate sparking sources from high concentration oxygen area. In addition, two fans were installed in the housing to ventilate oxygen in case there is a leak in the flow circuit and to lower system temperature.

*Design choice discussion*

An ideal MPR solution would maximize stored oxygen volume while minimizing the system’s physical footprint and operational complexity while being compatible with all commercial oxygen concentrators. The boost compressor and storage reservoir sub-components directly affect the MPR storage oxygen specification. Our solution set out to leverage off-the-shelf commercial products to capitalize on economies of scale costing and manufacturer component durability testing. In our solution, the MPR boost compressor needed to be sized so it was not choked by output specifications of oxygen concentrator and would reliably operate up to our maximum pressure without stalling.


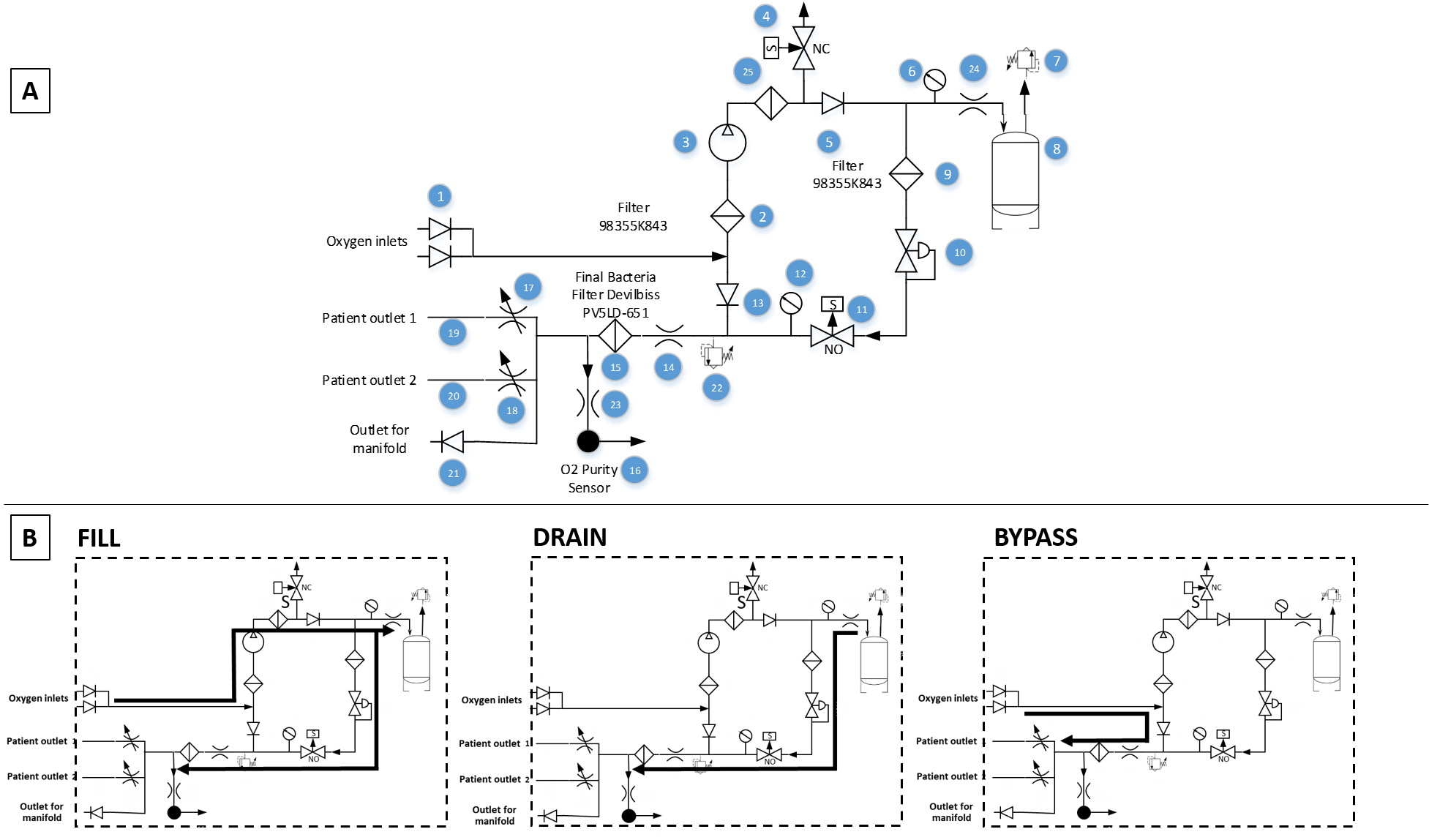


**Figure S1. Detailed schematic of MPR device. A.** Diagram illustrating valves, filters, oxygen inlets and outlets. Numbered components are described in Table S1. **B.** Functional states of the MPR: Fill, Drain, and Bypass.

**Table S1. Description of components of medium-pressure reservoir oxygen storage system**


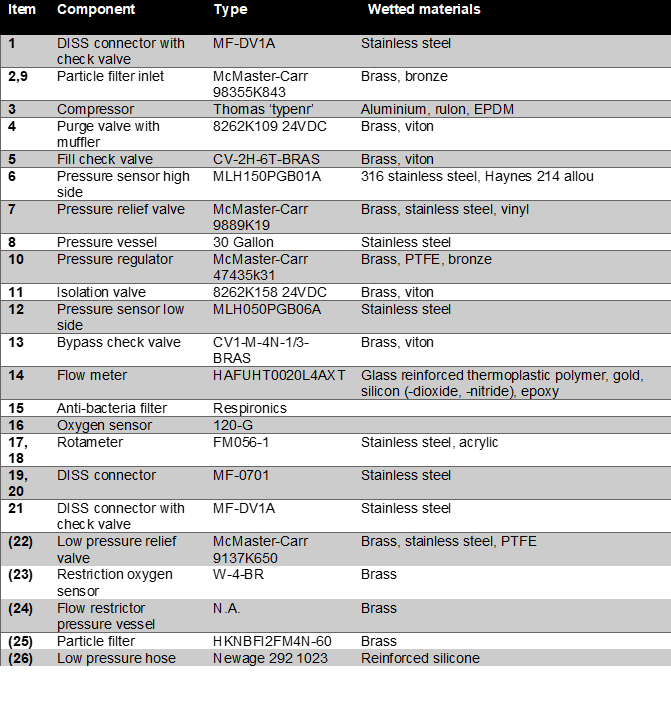


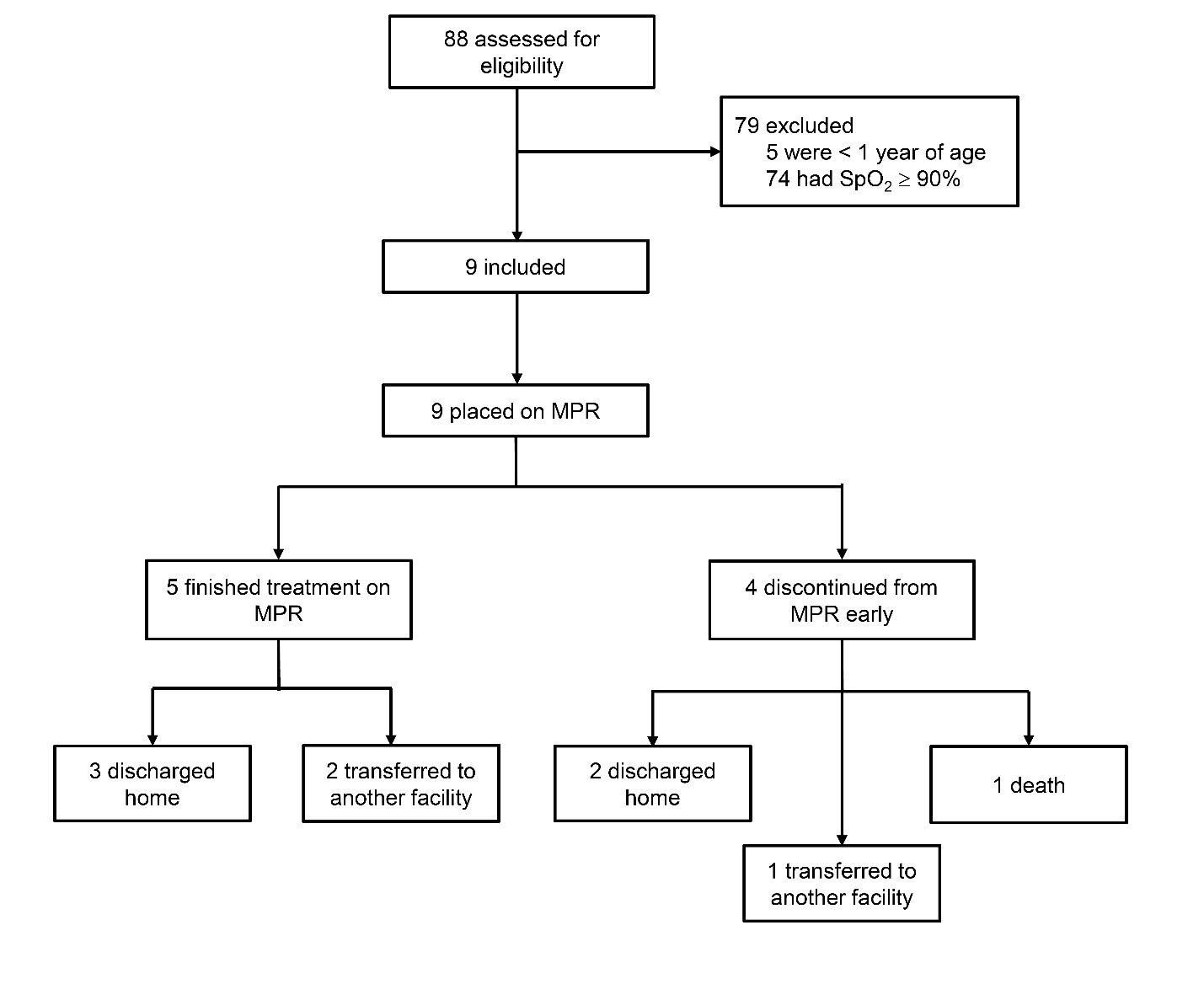


**Figure S2. Trial profile.** A total of 88 pediatric inpatients with acute respiratory illness were screened for hypoxemia. Nine patients met inclusion criteria and were administered oxygen using the MPR. Five completed their treatment on the MPR, whereas four patients were discontinued from the device.

**Supplemental References**

1. International A. ASTM G93 / G93M - 19, Standard Guide for Cleanliness Levels and Cleaning Methods for Materials and Equipment Used in Oxygen-Enriched Environments. 2019. Accessed August 19, 2020.
